# Genome Sequence of *Streptomyces toyocaensis* NRRL 15009, Producer of the Glycopeptide Antibiotic A47934

**DOI:** 10.1128/genomeA.00749-14

**Published:** 2014-07-31

**Authors:** Min Jung Kwun, Hee-Jeon Hong

**Affiliations:** Department of Biochemistry, University of Cambridge, Cambridge, United Kingdom

## Abstract

Here we report the draft genome sequence of *Streptomyces toyocaensis* strain NRRL 15009 which is the producer of the glycopeptide antibiotic A47934. The genome sequence is predicted to harbor a total of 26 secondary metabolite biosynthetic gene clusters including the A47934 cluster.

## GENOME ANNOUNCEMENT

*Streptomyces toyocaensis* strain NRRL 15009 was isolated from a sandy soil sample in the state of Washington, USA and described as the producer of A47934 (1). A47934 belongs to the glycopeptide family of antibiotics, an important class of antibiotics which also includes vancomycin and teicoplanin which are currently reserved in the clinic as the last-resort treatment of infectious disease caused by Gram-positive pathogens that are resistant to the antibiotics in mainstream use. A47934 is a sugarless glycopeptide antibiotic with a heptapeptide backbone structure closely related to that of teicoplanin, but is unique among reported glycopeptides as it contains a sulfate ester group. The complete sequence of the A47934 synthetic gene cluster in *S. toyocaensis* NRRL 15009 has previously been determined, and consists of 34 contiguous open reading frames (ORFs) encompassing 69 kb of chromosomal DNA (GenBank accession no. STU82965) (2). Here we present the first draft of the entire genome sequence of *S. toyocaensis* NRRL 15009.

The genome was sequenced to a coverage depth of 53-fold using a 454 GS-FLX instrument (Roche) with 454 titanium chemistry, and assembled using Newbler v2.5.3. This produced 87 contigs from 1,110,442 reads, and a total estimated genomic size of 7,341,583 bp. The genome has a G+C content of 72.04%. The average contig size is 97,848 bp and the largest contig size is 891,334 bp. Genome annotation was performed by the Prokaryotic Genome Annotation Pipeline (PGAP) with GeneMarkS+ (3, 4), and identified 6,315 coding sequence (CDS) regions, 82 pseudogenes, and 65 tRNAs. A single copy of each 5S rRNA, 16S rRNA, and 23S rRNA were also predicted in the draft genome sequence. The biosynthetic cluster of A47934 is located at 254,157 bp to 323,457 bp in the draft genome (on contig 1). This region also includes genes (*vanRst*, *vanSst*, *vanHst*, *vanAst*, *vanXst*, *murX*, *staO*, and *staP*) required for conferring self-resistance to the A47934 antibiotic. Using antiSMASH (5), a further 25 biosynthetic clusters potentially capable of producing secondary metabolites were also identified. These include three predicted to encode production of lantipeptides, two bacteriocin clusters, two siderophore clusters, three butyrolactone clusters, four nonribosomal peptide synthease (NRPS) clusters, four clusters encoding terpenes, a phenazine cluster, the ectoine biosynthesis cluster, and one type II-polyketide synthease (PKS) cluster.

The availability of the *S. toyocaensis* NRRL 15009 genome sequence will consequently assist with the search for new bioactive compounds of microbial origin, and will facilitate studies into resistance mechanisms and the regulation of secondary metabolite production.

### Nucleotide sequence accession numbers.

The draft genome sequence of *S. toyocaensis* NRRL 15009 has been deposited in the DDBJ/EMBL/GeneBank database under the accession no. JFCB00000000. The version described in this paper is version JFCB01000000.
